# The Time-Course of Changes in Muscle Mass, Architecture and Power During 6 Weeks of Plyometric Training

**DOI:** 10.3389/fphys.2020.00946

**Published:** 2020-08-04

**Authors:** Elena Monti, Martino V. Franchi, Francesca Badiali, Jonathan I. Quinlan, Stefano Longo, Marco V. Narici

**Affiliations:** ^1^Institute of Physiology, Department of Biomedical Sciences, University of Padua, Padua, Italy; ^2^MRC-ARUK Centre for Musculoskeletal Ageing, University of Nottingham, Derby, United Kingdom; ^3^School of Sport, Exercise and Rehabilitation Sciences, University of Birmingham, Birmingham, United Kingdom; ^4^NIHR Birmingham Biomedical Research Centre, University Hospitals Birmingham, NHS Foundation Trust and University of Birmingham, Birmingham, United Kingdom; ^5^Department of Biomedical Sciences for Health, University of Milan, Milan, Italy; ^6^CIR-Myo Myology Centre, Department of Biomedical Sciences, University of Padua, Padua, Italy

**Keywords:** skeletal muscle, muscle volume, quadriceps cross-sectional area, muscle power, fascicle length, muscle hypertrophy

## Abstract

**Purpose:**

To investigate the time-course of changes in knee-extensors muscle mass, architecture and function in response to plyometric training (PLT) performed on a novel training device, the Tramp-Trainer. This machine consists in a trampoline connected to an inclined sledge which allows the performance of repeated jumps while the subject is sitting on a chair.

**Methods:**

Eight healthy males (173.6 ± 4.7 cm, 69.7 ± 13.5 kg, 25.3 ± 4.6 years) underwent 6 weeks of bilateral PLT on the tramp-trainer machine. Training was performed three times per week (between 120 and 150 bounces per session). Knee-extensor maximum voluntary torque (MVT) and power, quadriceps femoris (QF) volume (VOL), cross-sectional area from the 20% to the 60% of femur length and CSA_*mean*_, together with vastus lateralis (VL) architecture (fascicle length, Lf, and pennation angle, PA) were assessed after 2, 4, and 6 weeks of PLT.

**Results:**

All results are presented as changes versus baseline values. MVT increased by 17.8% (week 2, *p* < 0.001) and 22.2% (week 4, *p* < 0.01), respectively, and declined to 13.3% (*p* < 0.05) at week 6 of PLT. Power increased by 18.2% (week 4, *p* < 0.05) and 19.7% (week 6, *p* < 0.05). QF VOL increased by 4.7% (week 4, *p* < 0.05) and 5.8% (week 6, *p* < 0.01); VL VOL increased by 5.2%, (*p* < 0.05), 8.2%, (*p* < 0.01), and 9.6% (*p* < 0.05) at weeks 2, 4, and 6, respectively. An increase in Lf was detected already at wk 2 (2.2%, *p* < 0.05), with further increase at 4 and 6 weeks of PLT (4 and 4.4%, respectively, *p* < 0.01). PA increased by 5.8% (*p* < 0.05) at week 6. Significant positive correlations were found between CSA_*mean*_ and Power (*R*^2^ = 0.46, *p* < 0.001) and between QF VOL and Power (*R*^2^ = 0.44, *p* < 0.024).

**Conclusions:**

PLT induced rapid increases in muscle volume, fascicle length, pennation angle, torque and power in healthy younger adults. Notably, changes in VL VOL and Lf were detectable already after 2 weeks, followed by increases in knee extensors VOL and power from week 4 of PLT. Since the increase in CSA_*mean*_ and QF VOL cannot fully explain the increment in muscle power, it is likely that other factors (such as adaptations in neural drive or tendon mechanical properties) may have contributed to such fucntional changes.

## Introduction

Muscle power is a major contributor of performance both in sports and in daily life activities, and several studies have been carried out in order to understand how power could be improved in young, older, and clinical populations (Harries et al., 2012; Cook et al., 2013; Reid et al., 2015). In this regard, plyometric exercise has been shown to be particularly effective for improving muscular performance both in young athletes (Ramìrez-Campillo et al., 2014) and recreationally active people (Makaruk et al., 2011). During plyometric exercise, a physiological phenomenon called “stretch-shortening cycle” (SSC) is naturally occurring (Ishikawa and Komi, 2004; Taube et al., 2012). SSC is characterized by a deceleration of the body (where the agonist muscle-tendon unit -MTU- is stretched) followed immediately by an acceleration in the opposite direction (where the same MTU is rapidly shortened) (Komi, 1984). The SSC enhances the ability of the neuromuscular system to produce force in a brief amount of time, thus coupling the two major contributors to muscle power (i.e., muscle strength and contraction velocity) (Cardinale et al., 2011).

Increments in maximum isometric and explosive strength have been reported after plyometric training (PLT) protocols (de Villarreal et al., 2008; Behrens et al., 2016). Over a training period, the contributing factors to such increases can be found in (i) muscle morphological adaptations (i.e., architectural, whole muscle and muscle fibers size changes) (Pottiger Jeffrey et al., 1999; Blazevich et al., 2003; Malisoux et al., 2006b; McKinlay et al., 2012), (ii) tendon and joint stiffness properties (Hirayama et al., 2017), and (iii) modifications of neural activation (Behrens et al., 2014, 2016; Hirayama et al., 2017).

Although previous studies investigated the changes of skeletal muscle size, strength and power following plyometric training programs, to the present date and to the best of our knowledge, none of these studies focused on the time-course of the muscular adaptations to PLT. Defining when and how these changes occur may be beneficial in order to plan time-efficient protocols in healthy or clinical populations. In fact, when programming a training protocol aiming to elicit specific muscular adaptations (such as increases in explosive strength and power for athletes, or recovery of these qualities after injury) it is of extreme importance to know the timing and amplitude of such adaptations in order to plan personalized and efficient intervetions, with a well-defined progression of the load based on truly achievable goals.

However, one important aspect of the plyometric training methods reported in literature is that the majority of the studies exploited free-body plyometric exercise programs using different intensities, workloads, durations, number of sessions per week, and unilateral or bilateral modalities (Behrens et al., 2014; Piirainen et al., 2014; Hirayama et al., 2017). It follows that the guidelines for optimal PLT workloads are still unclear regarding the adaptations in muscle hypertrophy and function (Davies et al., 2015). Indeed, although specific laboratory tools (such as force platforms) may be useful to standardize PLT parameters, these tools are expensive and not always available where athletes train, nor at gyms and/or facilities where healthy and clinical population would carry out specific rehabilitation programs. Therefore, the use of easy accessible and user-friendly devices may be ideal for standardizing the work performed and the training intensties. In the present study we employed the tramp-trainer machine (FREI AG, Hinterzarten, Germany, EU) consisting of a trampoline attached to an inclined sledge, enabling the performance of repeated plyometric jumps while the subject is sitting on a chair with the back fully supported. With this device, exercise is performed seated and with a defined trajectory, which helps to standardize the exercise movement between different subjects. The trampoline offers to exercise on a compliant surface, and while being different from the stiffer ground exploited in the majority of the classical plyometric trainings, this is highly suitable for a wide range of users, from athletes during rehab, to healthy people, and even elderly frail populations (Franchi et al., 2019).

Hence, the aim of the present study was to investigate the time-course of knee-extensors changes in muscle size, architecture and function in response to a 6-week plyometric training performed on the tramp-trainer device. Based on previous reports that show that early adaptations in muscle architecture were observed with resistance exercise (Seynnes et al., 2007), our hypothesis was that changes in muscle architecture would characterize the early responses to plyometric training and that modifications in muscle size (i.e., cross-sectional area -CSA- and volume) and architecture would accompain increases in muscle torque and power.

## Materials and Methods

### Participants

Fourteen young males were recruited to undergo a 6-weeks PLT program (height = 176.1 ± 6.3 cm, mass = 72.2 ± 13.8 kg, age = 25.4 ± 3.5 years). The pre-to-post adaptations to PLT (week 0 and week 6) are described in a previous study from our laboratory (Franchi et al., 2019). Among the fourteen participants, eight were randomly selected (height = 173.6 ± 4.7 cm, mass = 69.7 ± 13.5 kg, age = 25.3 ± 4.6 years; values are presented as mean ± SD) to undergo the investigation of the time-course of morpho-functional assessments, which results are shown in this manuscript. All volunteers were healthy, fully independent and recreationally active; they had not performed any plyometric or heavy strength training within the past 6 months, and they were asked to not practice any other kind of physical activity during the study. During the 6-weeks of PLT, subjects were required to not change their lifestyle and recreational activity habits in comparison to the pre-training situation. All the subjects were medically screened by means of a medical questionnaire, to exclude sufferers of joint disease and metabolic, respiratory or cardiovascular impairments. All subjects provided written, informed consent. This study was approved by The University of Nottingham Ethics Committee and was compliant with the Declaration of Helsinki.

### Trampoline-Trainer Exercise

**FIGURE 1:**
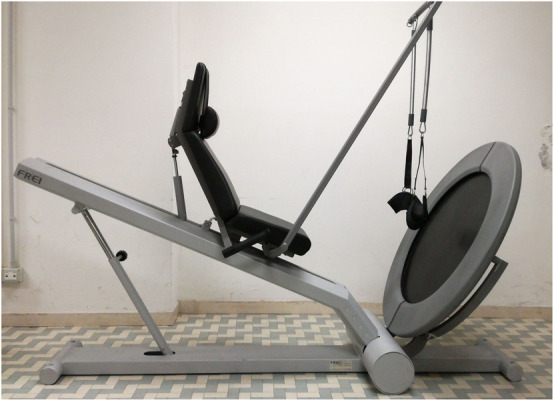
Tramp Trainer exercise device.

Training was performed on the “Trampoline-Trainer” (tramp-trainer) exercise machine (Figure 1) (FREI AG, Hinterzarten, Germany, EU). As previously described (Franchi et al., 2019), the tramp-trainer is a device made up by an inclined trampoline connected to a 1.5 m inclined sledge. The user is seated on a movable chair attached to the sledge and is required to flex and extend the lower limbs on the trampoline against his own body weight. The legs are supported by two ankle braces connected to a spring, as shown in Figure 1. Briefly, the exercise can be compared, to a certain extent, to a leg press movement, where the workload is represented by the body mass and the inclination of the carriage. In order to elicit the greatest possible response to exercise, carriage inclination was set at the maximum angle allowed by the machine (22°) and kept constant for all the training sessions.

Volunteers exercising on the tramp-trainer start from a semi-squat position with their knees flexed to a range between 90 and 80 deg (counting 0 deg as anatomical zero/full leg extension). After a maximal push of the lower limb muscles (hip extensors, knee extensors, and plantar flexors), the body and chair are displaced along the rail, followed by a landing on the elastic trampoline and an immediate recoil as the subject bounces back off the trampoline, and the entire cycle is then repeated for successive jumps (Franchi et al., 2019). Thus, the movement can be also compared to a drop jump, with the evident differences of (i) the inclined falling trajectory and (ii) the compliant surface where the landing and bouncing actions are performed.

### Training Protocol and 30 Repetition Maximum (RM) Assessment

Prior to the first training session, on a separate day, participants were familiarized with the tramp-trainer, being asked to perform a maximum of only 5 submaximal bounces. During such familiarization session, each participant practiced starting and landing from a knee angle of ca. 90° measured with manual goniometer (full knee extension was taken as anatomical 0).

Training was performed 3 times per week (on the same days and at the same time for each session) for a period of 6 consecutive weeks. At least one resting day between training sessions was programmed. Training volume was based upon the guidelines of Chu (1998), who recommended that beginner and intermediate status athletes should not exceed 120 foot contacts per session when implementing a new PLT program. Training volume was set at 4 × 30 repetitions for the first 4 weeks, followed by 5 × 30 repetitions for the final 2 weeks. The 30 jumps were preformed consequently, while rest between sets was 120 s. The total duration of the training session was about 20–25 min (for the first 4 and the last 2 weeks, respectively). Before the beginning of the training session, a short warm up consisting in 10 squats was performed.

Training load was matched across subjects by determining 30RM values before the start of the program. 30RM is the training level at which subjects could perform no more than 30 repetitions without a decrease in bounce performance. To quantify bounce performance, a meter ruler was attached to the carriage of the training device to monitor, via an operator visual inspection, average bounce height during the assessment. Subjects were instructed to bounce at their 30RM, and if they fell significantly below their 30RM height for up to 3 consecutive bounces, they were prompted to increase bounce height in order to maintain a constant training load. A significant decrease was determined as 5 cm or more below the 30RM height.

As training progressed, loading increased progressively because the velocity of impact increased due to higher bounce distances. As bounce distance increased due to training adaptations, new 30RM values were identified and implemented to progressively increase the training load. 30RM values were re-checked every 7 days (before the start of the first session each new training week). When volunteers reached the highest value of the meter ruler attached to the carriage (i.e., the top of the carriage) for most (∼90%) of the repetitions in a single training session, a 15 kg weighted vest was provided from the successive session in order to further increase the training load. In addition, training load increase was provided by increasing the number of series from week 4 of the training period.

During each training session a red mark was placed on the side of the inclined plane rail-track as a visual target corresponding to a knee flexion of ca. 90°.

### Functional and Morphological Parameters

To assess the time-course of training adaptations, test-sessions were performed before the beginning of the training (baseline), and after 2, 4, and 6 weeks of training. Testing sessions were scheduled right before the first training of the week, every 2 weeks, to allow a time lag of at least 48 h between the last training bout of the previous week and the testing day, in order to minimize exercise-induced edema. During all the test-sessions the following parameters were assessed: knee extensors isometric maximum voluntary contraction torque (MVT) and peak power production; vastus lateralis (VL) and quadriceps femoris muscle (QF) cross-sectional area (CSA) from the 20% to the 60% of the femur length, from which VL and QF volume were then estimated (see specific section below); VL muscle architecture at the 50% of the femur length. During the testing sessions, all participants were firstly scanned for US (VL architecture and QF CSAs) and afterward were randomly assigned to the following sequences (i) MVT testing first and power testing after (*n* = 4) or (ii) power testing first and MVT testing after (*n* = 4).

One week before baseline data collection -in the same day and time of the baseline session-, in 6 out of 8 subjects VL muscle architecture and QF and VL CSA were assessed, in order to evaluate the repeatability of the ultrasound operator through the Intraclass Correlation Coefficient (ICC) calculation.

#### Isometric MVT Testing

Isometric maximum voluntary torque was measured using an isokinetic dynamometer (Cybex Norm, Cybex International Inc., NY, United States) at a fixed joint angle of 70°, with full knee extension corresponding to 0°. The dynamometer had been previously calibrated following the manufacturer instructions, and gravity correction had been performed. After a brief warm-up, consisting of 10 short sub-maximal contractions at the 50% of MVT (1 s each), participants performed two maximum voluntary contractions, which lasted for 4 s, with a rest period of 30 s between contractions. Volunteers were provided with both real time visual feedback of torque production and a strong verbal encouragement during isometric contractions. The maximum isometric torque value (MVT peak) was chosen for data analysis.

#### Leg Extensor Power Testing

Knee extensors power was assessed using the Nottingham Power Rig (Nottingham University, Nottingham, United Kingdom). The power rig provides a measure of peak power during a hip and knee extension push againts a pedal (Bassey et al., 1992). Briefly, the device consist of a seat, and a lever (on which the feet are placed in order to exert force) which is connected to a flywheel by a chain. The leg extension movement is completed in 0.25–0.40 s. The resistance is minimal and remains nearly constant throughout the whole movement. On the testing day, participants were asked to push with both limbs as hard and as fast as possible (i.e., at their maximum velocity) on the raised foot plates through the full range of movement, and they were provided with strong verbal encouragement. Volunteers were required to perform a minimum of 5 repetitions of the leg-extension movement. If, within the 5 repetitions, a plateau was reached (i.e., the participant did not improve his performance from the 4th to the 5th repetition), the best value among the 5 trials was choosen for analysis. Conversely, if a progressive improvement in power was observed from the 4th repetition, unlimited efforts were allowed until a maximal power value was reached, and such value was considered for analysis. Participants were familiarized with the Nottingham Power Rig device, as at the first visit to the laboratory (prior to the first tesing session) they were asked to perform the exact protocol used for testing.

#### Muscle Cross-Sectional Area and Volume/Mass Calculation

**FIGURE 2:**
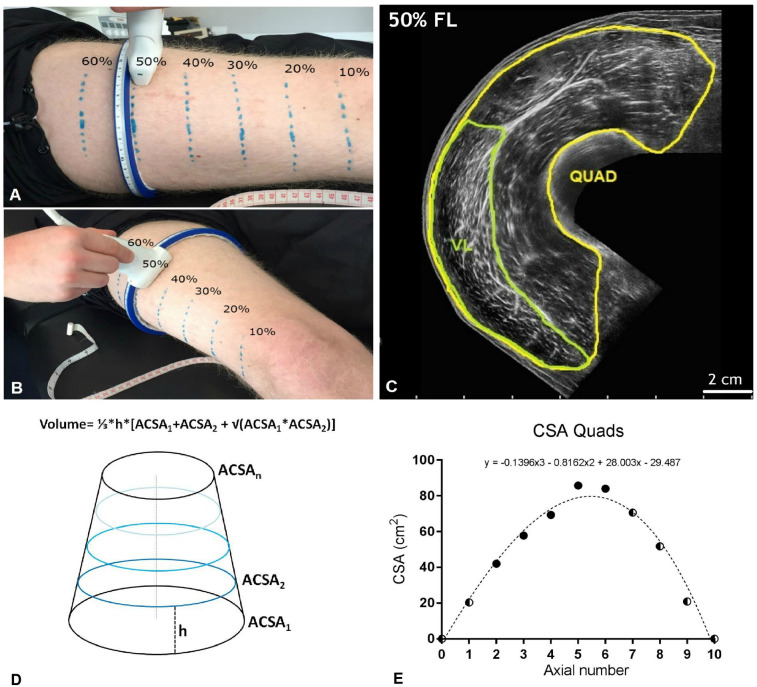
**(A,B)** Ulatrsonographic assessment of quadriceps femoris (QF) cross-sectional area (CSA) at different percentages of the femur length (measured from the great throcanter to the mid-patellar point). Region of interests considered were 20, 30, 40, 50, and 60% of the femur length. **(C)** Quad and vastus lateralis (VL) CSA obtained at the 50% of the femur length from a representative subject. **(D)** Truncated cone formula. The muscle volume between each couple of contiguos axials was calculated as 1/3*h*[ACSA_1_ + ACSA_2_ + √(ACSA_1_*ACSA_2_)], where ACSA stands for anatomical cross-sectional area. **(E)** Mathematical estimation of the lacking Quad CSA (10, 70, 80, and 90% of the femur length). A third order polynomial curve (reported for a representative subject) was fitted through the CSA values that were truly measured. For simplicity, the CSA values corresponding to the 0% and the 100% of the femur length were set as 0. Half and half symbols represent the estimated CSAs.

Muscle CSA of the QF was measured *in vivo* using extended-field-of-view (EFOV) ultrasonography imaging (Mylab70, Esaote, Genoa, Italy). A 47 mm, 7.5 MHz linear array probe was used to obtain images at different muscle length percentages. Different region of interest were identified at the 20, 30, 40, 50, and 60% of the femur length (measured from greater trochanter to the mid patellar point) and marked on the skin (Figure 2). As shown in Figure 2, we considered the mid patellar point as the beginning of the QF and VL muscles (0%), and the great trochanter as their end (100%). The operator then positioned the probe transversally on the medial portion of the leg, thus starting the acquisition when the medial borders of the vastus medialis had been identified. The acquisition consisted in moving the transducer along the transverse plane and it was stopped after visualizing the lateral borders of the VL. An adjustable guide was used in each acquisition in order to keep the same transverse path. Care was taken in order to keep as constant as possible both pressure during all the image acquisition and acquisition velocity throughout the different testing sessions. Two CSA images per femur length percentage were acquired and used for analysis.

Quadriceps femoris and VL only CSAs were measured tracing the muscle borders using ImageJ image analysis software. Two measures per parameter were taken on each image, and the average of them was considered.

For simplicity, QF and VL muscle volume were estimated by using the truncated cone formula (shown in Figure 2), as previously reported (Tracy et al., 2003). Partial muscle volume values were calculated from the 20% to the 60% of the subjects’ femur length using the CSAs data obtained from EFOV ultrasound scans. As no EFOV scans were performed below the 20% and above the 60% of the femur length marks, the remaining CSAs values for the 10, 70, 80, and 90% axials were estimated by fitting a spline curve (third order polynomial) through the CSA values that were truly measured (Seynnes et al., 2008).

The values corresponding to the 0 and 100% of the femur length were set as 0 (Figure 2). The calculated muscle volume, was assumed to closely approximate to muscle mass since muscle density is 1.0597 g/cm (Mendez and Keys, 1960).

Quadriceps femoris and VL volume were calculated as the sum of the calculated VOL between each femur length measured (20–60%) or estimated (10%, 70–90%) CSAs. In addition, QF CSA_*mean*_ was calculated as the average value of the 10–90% femur length CSAs per each subject and time-point.

#### Muscle Architecture

Muscle architecture of the VL muscle was measured *in vivo* using B-mode ultrasonography (Mylab70, Esaote, Genoa, Italy). Ultrasound images were obtained on the right leg, while the participant was laying in supine position; the 50% of the femur length (measured from the great throcanther to the mid patellar point) was found and marked with a skin marker, and afterward the probe was orientated transversally and the medial and lateral borders of the VL were identified. The 50% of the distance between the VL borders was chosen for image acquisition. VL fascicle length (Lf) and pennation angle (PA) were measured using a 100 mm, 10–15 MHz, linear-array probe as described by Franchi et al. (2015): the probe was positioned over the belly of the VL, carefully adjusted to the fascicle plane while minimal pressure was applied. Three ultrasound scans were then analyzed using ImageJ image analysis software^1^. Briefly, fascicles were digitized using the segmented line tool (thus not neglecting any fascicle curvature within their visible portion). Then, fascicle length was determined through manual extrapolation (i.e., straight line tool used) of fibers and aponeuroses if a portion of the fascicle extended outside of the captured ultrasound image. Usually 2–3 fascicles were eveluated for each scan and their average value was taken as final Lf value. PA was measured at the intersection between the fascicles and the deep aponeurosis. An average of at least 3 PA measures per image was performed (Franchi et al., 2018).

### Total External Mechanical Work

The total external mechanical work (*W*_*ext*,_ kJ) performed throughout the study was calculated as previously described (Franchi et al., 2019). Individual *W*_*ext*_ was calculated from body mass (plus 15 kg if participants were wearing the weighted vest), TT inclination, and average height reached during each bounce performed in each session relative to the maximum height of the track/carriage (i.e., every bounce height was recorded via visual inspection of an operator for all repetitions of set in each training session).

For a single set of 30 bounces, *W*_*ext*_ was calculated as follows:

*W*_*ext*_ (kj) = *[(BM + VM)* × *g* × *(sin22)]* × (*1.265-h_*ex*_)* × *30*

where *BM* (kg) represents the subject body mass, *VM* (kg) the mass of the weighted vest, *g* is the gravitational acceleration (9.81 m⋅s^–2^) multiplied by the sine of the pre-set inclination of the track, 22°(which was kept constant throughout the whole study), *h*_*ex*_ (m) represents the average height reached by every bounce in the set (relative to the total length of the track, 1.265 m), and 30 is the number of bounces performed per set.

### Statistical Analysis

All data are presented as average values (and standard deviation).

A priori power analysis was performed using G^∗^Power 3.1.9.4., using muscle power data obtained from a pilot study conducted in our laboratory. Power analysis revealed an actual power of 0.825 when considering a sample size of 8, with effect size being 1.02 and α = 0.05.

All data met the normal distribution criteria, established on quantitative inspection by Shapiro–Wilk test. Repeatability of the measurements was tested for muscle mass (CSA) and architectural (Lf, PA) values via calculation of the Intraclass Correlation Coefficient (ICC) of the ultrasound operator performing all the data acquisition and analysis. ICC values were rated good to very good, and resulted as follows: QF CSA = 0.996 (CV = 0.01%); VL CSA = 0.993 (CV = 0.04%); VL PA = 0.962 (CV = 0.05%); VL Lf = 0.966 (CV = 0.09%).

In addition, basing on the same data used for the repeatability analysis (ICC), the minimum detectable change was calculated, following the approach suggested by Weir (2005). The percentage minimum detectable change values (i.e., the minimum percentage increase that would represent a real change, which would not be dictated by repeated measures errors) were as follows: QF CSA = 1.38% (±0.85 cm^2^); VL CSA = 1.84% (±0.39 cm^2^); VL PA = 1.93% (±0.35°); VL Lf = 1.38% (±0.11 cm).

Differences in the functional, morphological, and architectural muscle components of the study during the time-course (baseline vs. post 2, 4, and 6 weeks of training) were investigated using a one-way repeated measures analysis of variance (ANOVA). Tukey’s multiple comparisons test was used to identify significance between the different time points. Significance level was set at *p* < 0.05.

Linear relationships between the percentage changes of the morphological and functional parameters during the time-course of PLT were tested using the Pearson’s product-moment correlation coefficient (*r*). Linear regressions were also calculated and the coefficient of determination (*R*^2^) was obtained. The level of significance was set at *p* < 0.05.

GraphPad Prism software (version 8.0; GraphPad software Inc., San Diego, CA, United States) was used to perform all statistical and *post hoc* analysis.

## Results

**FIGURE 3:**
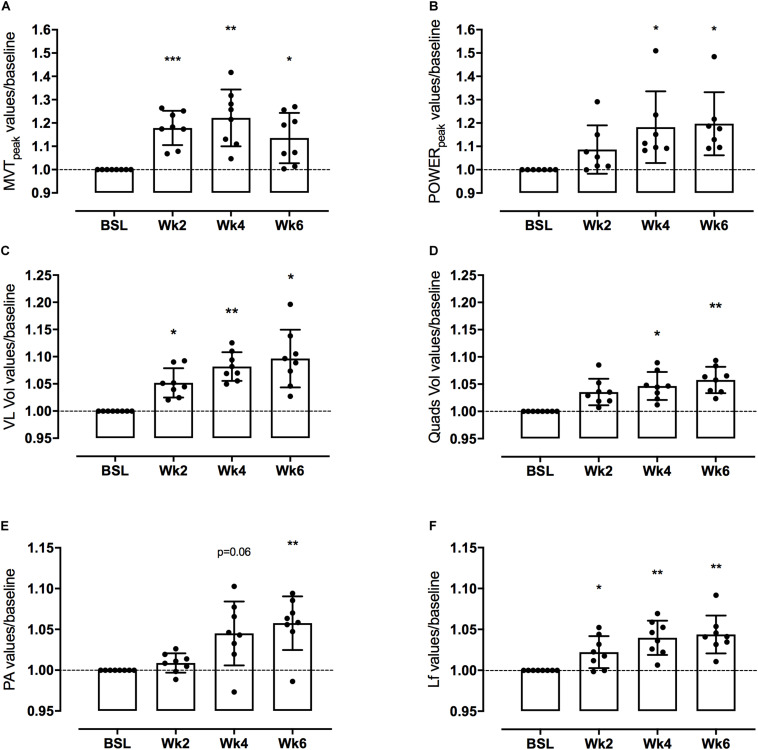
Knee extensor maximum voluntary torque (MVT) and leg extensor power (LEP) (**A,B**, respectively), Vsatus Lateralis (VL) and quadriceps (QF) volume (**C,D**, respectively), Vastus Lateralis (VL) Pennation Angle (PA) and Length of fascicles (Lf) (**E,F**, respectively) adaptations to a 6-week PLT measured after 2, 4, and 6 weeks. Changes are expressed in percentage vs. baseline (Δ%). Results are normalized for baseline values and shown as Means with scattered plots ± SD. *p < 0.05, **p < 0.01, ***p < 0.001.

**FIGURE 4:**
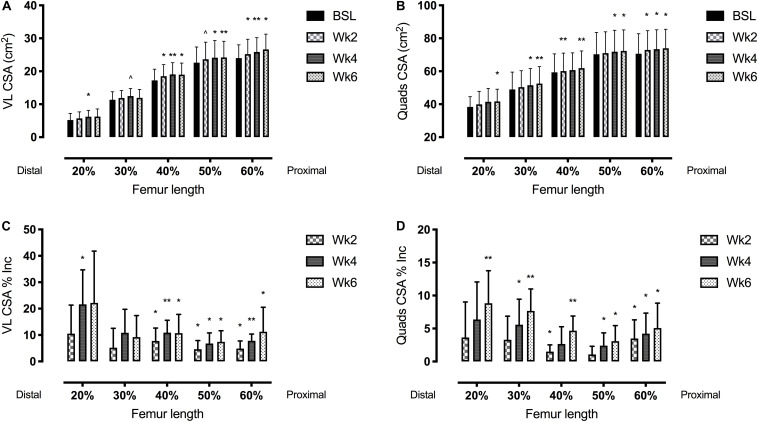
Vastus lateralis (VL) (left side, **A,C**) and quadriceps femoris (right side, **B,D**) muscle cross-sectional area (CSA) adaptations to a 6-week plyometric training measured after 2, 4, and 6 weeks. Top panels represent muscle CSA changes in absolute values (cm^2^). Bottom panels represent muscle CSA percentage changes (Δ%). Results are shown as Means ± SD. ^∧^*p* = 0.05, *p < 0.05, **p < 0.01.

The average values for functional and morphological adaptations at each time point are presented in Tables 1, 2 (means ± S.D), while the percentage changes of the same parameters during the time-course -compared to baseline- are shown in Figures 3, 4.

**TABLE 1:** Time-course of the muscle morphological and functional adaptations to a 6-week plyometric training.

	**Baseline**	**Week 2**	**Week 4**	**Week 6**	**ANOVA**
MVT (Nm)	230.37 ± 41.01	270.61 ± 46.98***	280.26 ± 52.46**	260.94 ± 51.06*	*F* = 13.69; *p* = 0.0004; *R*^2^ = 0.66
Power (W)	519.64 ± 104.12	564.83 ± 125.20	609.54 ± 113.36*	617.50 ± 110.02*	*F* = 11.42; *p* = 0.0042; *R*^2^ = 0.66
*W*_*Ext*_/session (kJ)	27.67 ± 4.63	30.79 ± 6.25	31.22 ± 5.96*	39.41 ± 5.85***	*F* = 52.03 *p* < 0.0001 *R*^2^ = 0.88
VL PA (deg)	16.50 ± 1.42	16.66 ± 1.56	17.27 ± 1.92	17.46 ± 1.73**	*F* = 14.03; *p* = 0.0030; *R*^2^ = 0.67
VL Lf (cm)	7.79 ± 1.13	7.95 ± 1.05*	8.02 ± 1.11**	8.13 ± 1.17**	*F* = 15.32; *p* = 0.0002; *R*^2^ = 0.69
QF CSA_*mean*_ (cm^2^)	49.95 ± 8.37	51.74 ± 8.77	52.28 ± 8.78*	52.80 ± 8.66**	*F* = 19.7; *p* = 0.0026; *R*^2^ = 0.77
QF volume (cm^3^)	1867.70 ± 363.03	1934.19 ± 383.46	1954.40 ± 385.11*	1974.14 ± 382.01**	*F* = 18.97; *p* = 0.0027; *R*^2^ = 0.76
VL volume (cm^3^)	593.22 ± 123.46	623.71 ± 133.32*	640.99 ± 133.40**	648.70 ± 131.85*	*F* = 14.91; *p* = 0.0016; *R*^2^ = 0.71

All the parameters were assessed at baseline and at 2-, 4-, and 6-week time points. MVT, maximum voluntary torque; LEP, leg extensors muscles power; W_*ext*_, external work; VL, vastus lateralis muscle; PA, pennation angle; Lf, fascicle length; QF, quadriceps femoris; CSA_*mean*_, average of the cross-sectional areas taken at the 20, 30, 40, 50, and 60% of the femur length with the CSAs estimated at the 10, 80, and 90%. Results shown as Means ± SD. VS Baseline *p < 0.05, **p < 0.01, ***p < 0.001.

**TABLE 2:** Time-course of the vastus lateralis (VL) and quadriceps femoris (QF) cross-sectional areas (CSA) adaptations to a 6-week plyometric training.

**VL CSA (cm^2^)**	**Baseline**	**Week 2**	**Week 4**	**Week 6**	**ANOVA**
20% FL	5.21 ± 2.01	5.69 ± 2.01	6.20 ± 1.93*	6.26 ± 2.32	*F* = 4.916; *p* = 0.0307; *R*^2^ = 0.50
30% FL	11.28 ± 2.64	11.78 ± 2.48	12.39 ± 2.50	12.24 ± 2.63	*F* = 8.602; *p* = 0.0096; *R*^2^ = 0.63
40% FL	17.24 ± 3.40	18.51 ± 3.51*	19.05 ± 3.55**	18.99 ± 3.49*	*F* = 15.48; *p* = 0.0006; *R*^2^ = 0.72
50% FL	22.61 ± 4.76	23.65 ± 5.19	24.12 ± 5.23*	24.2 ± 4.91**	*F* = 14.55; *p* = 0.0016; *R*^2^ = 0.71
60% FL	24.0 ± 4.04	25.19 ± 4.52*	25.86 ± 4.34***	26.65 ± 4.61*	*F* = 7.702; *p* = 0.0231; *R*^2^ = 0.56

**QF CSA (cm^2^)**	**Baseline**	**Week 2**	**Week 4**	**Week 6**	**ANOVA**

20% FL	37.79 ± 6.47	39.37 ± 8.44	40.33 ± 8.20	41.21 ± 7.96*	*F* = 6.56; *p* = 0.0211; *R*^2^ = 0.57
30% FL	48.98 ± 10.52	50.41 ± 9.91	51.51 ± 10.15*	52.53 ± 10.30***	*F* = 18.51; *p* ≤ 0.0001; *R*^2^ = 0.76
40% FL	59.33 ± 11.20	60.14 ± 10.88**	60.74 ± 10.48	61.91 ± 10.42**	*F* = 13.26; *p* = 0.0006; *R*^2^ = 0.69
50% FL	70.34 ± 10.21	71.01 ± 12.96	71.90 ± 12.94*	72.35 ± 12.74*	*F* = 11.56; *p* = 0.0030; *R*^2^ = 0.66
60% FL	70.61 ± 12.16	72.92 ± 11.70*	73.42 ± 11.77*	73.96 ± 11.50*	*F* = 14.41; *p* = 0.0060; *R*^2^ = 0.71

CSAs are taken at the 20, 30, 40, 50, and 60% of the femur length (FL). Results shown as Means ± SD. VS Baseline *p < 0.05, **p < 0.01, ***p < 0.001.

### Time Course of Changes in Muscle Function

Mean MVT values increased by 17.8 ± 7.4% (*p* < 0.001) after only 2 weeks of PLT. After 4 weeks, MVT further increased (n.s. vs. week 2) reaching a peak percentage increment by 22.2 ± 12.2% compared to baseline (*p* < 0.01) (Figure 3). In contrast, post-training tests revealed a decrease in MVT values from week 4 to week 6 (n.s.); nonetheless pre-to-post MVT remained significantly different, presenting a total increase by 13.5 ± 10.8% (*p* < 0.05).

Power increased by 18.2 ± 15.4% (*p* < 0.05) after 4 weeks of PLT, reaching a final pre-to-post increment of 19.7 ± 13.5% (*p* < 0.05 vs. baseline) (Figure 3).

### Time Course of Changes of QF and VL CSA and Volume

The time course of changes in muscle size is reported in Table 2 and Figures 3, 4.

Quadriceps femoris CSA showed significant adaptations after 2 weeks of PLT at 40 and 60% of the femur length, increasing, respectively, by 1.5 ± 1.0% and 3.5 ± 2.8% (*p* < 0.05). Similarly, VL CSA taken at the 40% and the 60% of the femur length increased, respectively, by 7.8 ± 4.9% and 4.9 ± 2.9% (*p* < 0.05) after 2 weeks of PLT.

From 4 weeks onwards, all the QF CSAs (except the one taken at the 20% of the femur length) showed a significant increment compared to baseline (at 20%: 5.6 ± 3.9%, *p* < 0.05; at 30%: 2.7 ± 2.6%, *p* < 0.05; at 50%: 2.4 ± 1.9%, *p* < 0.05; and at 60%: 4.2 ± 3.1%, *p* < 0.05). At 6 weeks, all QF CSAs were statistically larger compared to baseline (at 20%: 8.8 ± 4.9%, *p* < 0.05; at 30%: 7.7 ± 3.3%, *p* < 0.001; at 40%: 4.7 ± 2.2%, *p* < 0.01; at 50%: 3.1 ± 2.4%, *p* < 0.05; and at 60%: 5.1 ± 3.8%, *p* < 0.05).

Conversely, VL CSA increased only at the 40, 50, and 60% of the femur length after 4 (*p* < 0.05) and 6 weeks (*p* < 0.05) of PLT compared to baseline. Specifically, at 4 and 6 weeks, the percentage increment vs. baseline were, respectively: 10.9 ± 4.7% (*p* < 0.01) and 10.7 ± 7.1% (*p* < 0.05) for the 40%; 6.8 ± 4.0% (*p* < 0.05) and 7.4 ± 4.2% (*p* < 0.01) for the 50%; and 7.8 ± 2.6% (*p* < 0.001) and 11.2 ± 9.3% (*p* < 0.05) for the 60% of the femur length. Although, at week 6, VL CSA at the 20 and 30% of femur length showed a marked increase, this was not statistically significant (22.2 ± 19.6%, *p* = 0.11; and 9.2 ± 8.2%, *p* = 0.07; respectively).

Quadriceps femoris CSA_*mean*_ showed a progressive increment by 4.7 ± 2.8% at 4 weeks (*p* < 0.05 vs. baseline) and by 5.8 ± 2.7% at 6 weeks (*p* < 0.01 vs. baseline).

Quadriceps femoris volume increased by 4.7 ± 2.8% (*p* < 0.05) and 5.8 ± 2.6% (*p* < 0.01) after 4 and 6 weeks of PLT, respectively, compared to baseline. Compared to baseline, VL volume increased significantly by 5.2 ± 2.9% (*p* < 0.05) after only 2 weeks of PLT, by 8.2 ± 2.9% (*p* < 0.01) after 4 weeks, reaching an increment by 9.6 ± 5.7% (*p* < 0.05) at 6 weeks.

### Time Course of VL Muscle Architecture Changes

Vastus lateralis Lf showed a significant increment by 2.2 ± 2.0% already after 2 weeks of training (*p* < 0.05), thereafter increasing linearly up to 4.0 ± 2.1% at week 4 compared to baseline (*p* < 0.01 vs. baseline and *p* < 0.05 vs. week 2) and by a further 0.4 ± 2.3% at week 6 (n.s. vs. week 4), reaching a final pre-to-post training increase by 4.4 ± 2.3% (*p* < 0.01) (Figure 3).

Vastus lateralis PA, instead, presented a significant increase only after 6 weeks of PLT (*p* < 0.01) by 5.8 ± 3.3% compared to baseline (Figure 3).

### Correlations Between Changes in Muscle Function and Muscle Morphology

Significant correlations were found between training induced-changes in CSA_*mean*_ and Power (*R*^2^ = 0.46, *p* < 0.001) as well as QF VOL and Power (*R*^2^ = 0.44, *p* < 0.024).

None of the training induced-changes in muscle architecutre parameters (PA, Lf) were significantly correlated with the increase in Power.

### Total External Mechanical Work

The total external mechanical work performed after the first training session (27 ± 4.6 kJ) was significantly different from the first session of the 4th week (31.2 ± 6 kJ, *P* < 0.05) and of the 6th (39.4 ± 5.9 kJ, *p* < 0.001) (Table 1). The average total work performed throughout the whole training period was 606.1 ± 124.6 kJ.

## Discussion

In the present study we investigated the time-course of changes in muscle mass, architecture and function in response to 6-week PLT performed on the tramp-trainer machine.

At the end of the training period, knee-extensors power was found to be increased by 19.7%, and this was accompanied by increments in both QF and VL volume and CSA_*mean*_. In addition, VL Lf and PA increased by 4.4 and 5.8%, respectively.

Notably, early significant changes in muscle function (MVT), architecture (Lf) and morphology (VL and QF CSA, VL volume) were detectable after only 2 weeks of PLT. Among these changes, VL CSAs were found to be increased especially in muscle regions closer to the mid-belly, and a similar trend was observed for QF CSA. Furthermore, an increase in knee-extensors power (18.2%) was found just after 4 weeks of PLT, together with significant changes in QF volume (4.7%). Changes in QF volume significantly correlated with the increase in muscle power (*R*^2^ = 0.44).

### Adaptations in Muscle Function: Muscle Strength

The 6-week PLT intervention was highly effective for increasing knee-extensors MVT.

Consistent with previous studies investigating strength adaptations to training, the increase in MVT was very rapid, reaching 17.8% after just 2 weeks of training. A small portion of this increase can be explained by muscle hypertrophy (3.5% increase in QF CSA_*mean*_), while it can be speculated that the main increment may be ascribable to an increased neural drive known to account for the early gain in muscle strength/power with resistance training (Narici et al., 1989; Häkkinen et al., 2001; Aagaard et al., 2002; Seynnes et al., 2007). As the training period progressed, MVT further increased to 22.2% at week 4 but no further increase was seen thereafter; in fact, at 6 weeks, the increase in MVT (13.3%) was 61% that of week 4. The slight decrement of MVT in the last 2 weeks of training is most likely due to a loading limitation (i.e., the training-load adjustments during the 6 weeks of PLT) of the plyometric training device used in this study. Indeed, as the participants bouncing performance improved, a 15 kg weighted vest was worn by the subjects to increase muscle loading, but as performance further improved at week 4, extra series were added to increase training volume. It seems thus that the combination of the 15 kg weighted vest plus the extra bounces series was not sufficient to provide enough muscle loading to achieve further gains in muscle strength after 4 weeks of training.

In addition, as gains in muscle strength appear to be training-specific (Tillin and Folland, 2014), another possible explanation could be related to the specificity of the movement used during plyometric training. In fact, this may have not been specific in relation to the one required to develop the maximal torque on the dynamometer device (i.e., isometric knee extension). Furthermore, only one joint angle (70°) was used to measure MVT throughout the whole study. Thus, we cannot exclude that a shift in the angle-torque relationship may have occurred in some volunteers as a resultant of more pronounced changes in muscle architectural features (Reeves et al., 2004). This would have in turn affected the force developed at a single joint angle (i.e., hence the 4-to-6 weeks drop in MVT).

The findings related to the increments in power and strength appear consistent with previous work, showing that exploiting combinations of countermovement jumps, drop-jumps, squat jumps and jumps using sledge apparatus would elicit enhancements in knee-extensors power and explosive force production (Pottiger Jeffrey et al., 1999; Fatouros et al., 2000; Kyröläinen et al., 2005; Piirainen et al., 2014). Some studies have shown that PLT could induce increases in knee-extensors maximum strength (Hakkinen et al., 1990; Fatouros et al., 2000; Malisoux et al., 2006b; Behrens et al., 2014), although others have reported no changes (Kyröläinen et al., 2005; Paleckis et al., 2015). The majority of these studies included PLT performed on stiff grounds, which are different from the compliant surface of the tramp-trainer. However, the similarities of such results with the one of the present study, together with reports that PLT protocols conducted in different conditions [such as in water (Martel et al., 2005; Robinson et al., 2004)] lead to similar increases in torque and power outputs, seem to suggest that the adaptations observed for muscle function are in line with those found after “classic” PLTs.

A second critical point when comparing the results found in the literature to the ones of the present study could be the difference in training volume. Indeed, our participants performed a high number of jumps per session (i.e., ∼120–150). In this regard, de Villarreal et al. (2008) investigated the differences in knee-extensor strength and countermovement jump performance between low, medium and high frequency and volume PLT, showing that functional gains were not influenced by such variables.

### Adaptations in Muscle Function: Muscle Power

It is noteworthy that, despite the lack of further gain in muscle torque, muscle power kept increasing till week 6.

Since power is the product of force and velocity, the increment of power may be expected to have been due to an increase in contraction speed. In this regard, a significant and progressive increase in fascicle length was found at all time points (2.2% at 2 weeks, 4.0% at 4 weeks and 4.4% at 6 weeks). A similar observation applies to CSA_*mean*_, which showed a progressive increment (3.5% at 2 weeks, 4.7% at 4 weeks and 5.8% at 6 weeks). Hence, consistent with the notion that volume is the product CSA_*mean*_ and muscle length (Jones and Round, 2000) both an increase in fascicle length (reflecting a potential increase in the number of sarcomeres in series) and in CSA_*mean*_ likely contributed to the increase power observed in this study. This seems confirmed by the significant correlation between the % changes of CSA_*mean*_ (*R*^2^ = 0.46, *p* < 0.001) and QF volume and power (*R*^2^ = 0.44, *p* < 0.024).

In addition to these morphological factors, we cannot exclude that neural or tendon adaptations may have contributed to the increased force and power outputs. Previous studies have shown an increase in neural drive of knee-extensors (Behrens et al., 2013) and plantar flexors (Kubo et al., 2007) during MVT (Behrens et al., 2016) following PLT. In contrast, Kyröläinen et al. (2005) found a significant increase in plantar flexors activation following PLT but no differences were observed for the knee extensors. Interestingly, a higher knee extensors pre-activation when performing vertical jumps after PLT has been previously reported (Kyrölänen et al., 1991). Similarly, Hirayama et al. (2017) reported neuromuscular activity alterations of both agonist and antagonist of plantar flexors during a SSC performance, indicating that increased power output could be related to changes in leg muscle activation strategies (or inter-muscular coordination). Thus, although no complete agreement regarding the specific neural adaptations to PLT has been reached in the literature, it could be speculated that training-specific adaptations could have occurred and contributed to the changes in muscle function reported in this study.

In addition, tendon properties (CSA, stiffness, and young’s modulus) are also known to be affected by the training-induced load (Bohm et al., 2015; Wiesinger et al., 2015; Kubo et al., 2017). Notwithstanding, the literature presents some controversial results when it comes to plyometric loading. Fouré et al. (2010) reported an increase in Achilles tendon stiffness after 14 weeks of PLT, and a similar result was found by Hirayama et al. (2017) after 12 weeks. On the other hand, Kubo et al. (2007) found an increase in ankle joint stiffness following 12 weeks of unilateral PLT, although no differences were found in Achilles tendon stiffness. This result was confirmed by a later study from the same authors (Kubo et al., 2017) that highlighted an increased active muscle stiffness with no changes in tendon CSA, stiffness and hysteresis following a 12-week PLT. The lack of changes in Achilles tendon CSA was also reported by Fouré et al. (2012) after 14 weeks training. The majority of the studies focusing on tendon adaptations to PLT have investigated the changes detectable in the Achilles tendon; to the best of our knowledge, only one study investigated the effects of drop-jumps training on patellar tendon so far (Paleckis et al., 2015), showing increases in CSA but not thickness of this tendon toward the end of the 9-days intervention protocols.

Taken together we cannot exclude that tendon adaptations may have occurred during the training period, but we cannot speculate toward which direction. Further research should aim to clarify this.

### Adaptations in Muscle Size

We have also investigated the distribution of morphological changes of the QF and VL muscles, in terms of muscle volume and CSA values collected at different sites of femur length (Figure 4).

Both VL and QF muscle volume increased linearly throughout the training period; however, VL volume increment was significant after only 2 weeks, contributing to QF volume changes which were detectable after 4 weeks of PLT, in accordance with the increases in knee-extensors power.

Interestingly, the majority of the works focusing on PLT interventions did not evaluate the possible muscle hypertrophic responses to such training modality. Two studies from Kubo et al. (2007, 2017) reported increased plantar flexors muscle volume or thickness (respectively) following 12 weeks of PLT; on the other hand, Fouré et al. (2012) reported unchanged gastrocnemius medialis CSA measured by ultrasound after 14 weeks of intervention. As for the quadriceps muscle, few authors (Pottiger Jeffrey et al., 1999; Malisoux et al., 2006a,b) reported hypertrophy of both slow and fast fibers of the VL following 8 weeks of intervention. McKinlay et al. (2012) reported an 8.1% increased VL muscle thickness after 8 weeks of PLT, and Váczi et al. (2014) found a 2.5% increase in quadriceps CSA measured via magnetic resonance after 10 weeks of intervention in elderly people. Very interestingly, Earp et al. (2015) trained for 8 weeks 18 subjects with squat jumps (9 with parallel thigh depth jumps and 9 with volitional depth jumps), and found increases in QF and VL CSA in the distal (33%) and mid (50%) sites of the muscles.

Our results also show that, consistently with the findings of Earp et al. (2015), the muscle volume adaptations were driven by specific regional hypertrophic responses, which appeared significant at different time points. The earliest changes in both VL and QF CSAs were detected in the mid-belly region (40–60% of femur length, after only 2 weeks of PLT) compared to the distal one (20–30% of the femur length, at week 4) (Figure 4). However, after 6 weeks, the highest percentage VL and QF CSA regional increments were reported distally.

The observed distal hypertrophy highlights the possible influence of the eccentric component of PLT. In fact, not only there is previous evidence of regional hypertrophy following different training modalities (Narici et al., 1989; Franchi et al., 2014), but also of a preferential distal growth of VL after eccentric exercise in comparison to a more marked mid-portion hypertrophy after concentric exercise (Franchi et al., 2014). Moreover, we previously reported regional differences in activation of mechanotransduction signaling (i.e., FAK, Meta-Vinculin), with the activation of such intergin-complex proteins being greater after pure eccentric training (Franchi et al., 2018). This could have induced greater potential for regional hypertrophic responses, as FAK has been shown to modulate muscle protein synthesis (Klossner et al., 2009). However, similar responses in muscle protein synthesis of mid muscle vs. distal sites were reported after 4 and 8 weeks of eccentric vs. concentric training (Franchi et al., 2015, 2018). Thus, such processes should be object of future investigations.

Regarding the significant changes in both VL and QF CSA after just 2 weeks of training (which in turn influence the changes in muscle volume), some authors have suggested that such early responses may be influenced by the edema provoked by the eccentric component of exercise in the first training sessions (Damas et al., 2018). The exact time point when an increase in CSA can be considered as true hypertrophy has been investigated by several studies and it is still currently under debate. DeFreitas et al. (2011) highlighted that 3-to-4 weeks of resistance training are sufficient to elicit muscle hypertrophy even when considering the potential bias introduced by muscle edema. Stock et al. (2017) investigated the hypertrophic adaptations to a concentric-only resistance training program, showing that after only 7 sessions (i.e., just over 2 weeks of training) significant hypertrophy could be detected. Furthermore, Wilkinson et al. (2014) demonstrated that cumulative myofibrillar protein synthesis is increased after only one bout of resistance training, peaking in day 2–4 post-exercise and then decreasing.

Lastly, in order to ensure our results not to be biased by the repeated measures error, we calculated the minimum detectable change, i.e., the minimum change required to assume that a real adaptation had occurred. For VL CSA, the minimal difference was found to be of 1.84%; within our subjects, only one of them showed increments lower than this at week 2 and week 4, and the average increases were at least 4.6% (week 2). For QF CSA, the minimal difference resulted of 1.38%; at week 2, 4 subjects presented increments higher than this value and 4 presented lower increments. However, the mean increment was higher than 1.38% (=minimum detectable change) in the CSAs taken at almost all the femur lengths, excluding the 50% (+1.06%) which resulted not significant. At weeks 4 and 6, all the subjects but 2 (week 4) and 1 (week 6) showed increments higher than 1.38%, and so did the average value calculated for all volunteers.

Thus, it is plausible that the observed adaptations in muscle size could be regarded as true hypertrophic responses.

### Adaptations in Muscle Architecture

We observed a significant increase in fascicle length after 2 (2.2%), 4 (4%), and 6 (4.4%) weeks of training, while PA showed an increment (5.8%) only at the 6-week time point. Similarly to the approach used to detect whether these small (albeit significant) changes could be regarded as true adaptations (i.e., not biased by the repeated measures error) we calculated the minimum detectable change for both Lf and PA. For VL Lf, the minimum detectable change was 1.38%; at week 2, 3 out of 8 subjects showed increments lower than this value, while the average was higher. At weeks 4 and 6 only 1 subject displayed increments lower than 1.38%. VL PA minimum detectable change was 1.93%, and this value was not reached in 6 out of 8 subjects at week 2 (and also by the average in this time point), and by 1 subject at weeks 4 and 6. Accordingly, PA was significantly improved after 6 weeks and showed a trend after 4 weeks.

To the best of our knowledge, only very few studies have considered muscle architecture with respect of PLT. Blazevich et al. (2003) reported an increase in Lf after 5 weeks of sprinting and jumping training, accompanied by a decrease in PA compared to baseline. However, participants had been performing resistance training for the 4 weeks preceding training intervention, and thus the decrease in PA could be also related to the lack of weights in the 5 weeks of sprint and jump intervention. Aeles et al. (2017) investigated, in a cross-section study, the differences in gastrocnemius medialis muscle architecture between jumping athletes and untrained controls, finding no differences in Lf but higher PA in athletes.

Similarly to Blazevich et al. (2003), our results show increases in VL Lf following PLT; this phenomenon is expected to reflect an increase in the number of sarcomeres in series, and thus in maximum shortening velocity (Bodine et al., 1982). Animal studies have shown that sarcomere addition can occur already after 5, 10, and 28 days in response to training modalities involving muscle lengthening (Lynn et al., 1998; Butterfield et al., 2005; Chen et al., 2020). However, recent studies in intact whole mice muscle have described some variability in sarcomere length along different muscle regions (Moo et al., 2016), which become even more non-uniform upon activation (Moo et al., 2017). Thus, we can speculate that changes in Lf may represent a result of both sarcomere addition and change in sarcomere operating length.

The mechanisms responsible for the increase in fascicle length are likely related to the fascicle stretch caused by the repeated SSC involved in plyometric training. These SSCs are thought to involve consecutive muscle shortening (concentric) and lengthening (eccentric) contractions, but, at a muscle level, the real eccentric nature of the deceleration phase during the SSC has been often debated. Theoretically, the muscle is firstly lengthened before it rapidly contracts concentrically (Bosco et al., 1981): however, some previous reports have suggested that the structures that stretch during the lengthening phase are the tendon complex and the series elastic components, while muscle fascicles behave “quasi isometrically” (Ishikawa and Komi, 2004). Nevertheless, these studies have been often conducted on the plantar-flexors MTU. Interestingly, two previous reports have observed, in addition to MTU and tendon lengthening, that VL fascicle lengthened during drop-jumps (Ishikawa, 2003) and countermovement jumps (Nikolaidou et al., 2017).

It follows that the mechanical stretch applied to the fascicles during training protocols involving a SSC may not be completely comparable to the one observed after pure eccentric contractions (Franchi et al., 2014). Notwithstanding, in the present study, during the landing-push off transition, volunteers were asked to maintain similar range of motion, corresponding to a maximum knee flexion of ca. 90°. In fact, previous work showed that isometric contractions at longer muscle lengths (at a knee joint angle similar to the one reached during bouncing in the present study) can lead to an increase in Lf (Noorkõiv et al., 2014). Lastly, we cannot exclude that the aponeuroses may have contributed to such adaptations in Lf: in fact, it has been shown that aponeurosis stiffness changes with muscle-tendon complex length (as would occur in the SCC), affecting fascicle strain (Raiteri et al., 2018). Fascicle stretch would in turn trigger mechanotransduction signaling pathways, which we have recently reported to be associated with changes in muscle architecture (Franchi et al., 2018).

### Limitations

We acknowledge that the present study has some limitations.

First of all, we did not recruit a control group. However, we recommended our participants to not change their habitual levels of recreational physical activity and to not introduce any new exercise modality in the training period. Second, we acknowledge that the small sample size allow us to draw preliminary conclusions. Furthermore, our participants were not trained individuals and were novice to this training modality: it follows that the responses observed could be influenced by this aspect. Indeed, the relatively small (albeit significant) changes in muscle mass and architecture, and the observed increments in MVT and power could be less pronounced if the present PLT protocol would have been applied to an already trained population.

In addition, there are limitations linked to the plyometric training device we used. This device enables to increase the work output both by increasing the slope of the inclined sledge as well as by increasing the distance covered during the push as muscle power increases. To maximize the load, we used the maximum chair slope that could be set in this device. As the subject power increases with the training progression, he eventually reaches the end of the rail track along which the seat travels. To increase load intensity and prevent the subject reaching the end of the rail track we overloaded the subject with a 15-kg weighted vest. However, after 4 weeks of training, most participants were nearing the end run again, so to add extra work-volume we increased the number of series of sets of 30 bounces. This procedure was undoubtedly effective for increasing the total work performed but the overloading was probably insufficient to induce a further gain in muscle force.

On the other hand, also the use of the 15-kg weighted jacket could represent a potential limitation. Indeed, this was certainly sufficient for ensuring a gain in muscle strength up to 4 weeks of training but not enough to produce further gains after 4 weeks. However, despite these limitations, power output progressively increased up to the end of training period.

Lastly, we did not investigate either neural or tendon adaptations to the training protocol used (for a detailed discussion, see section “Adaptations in Muscle Function” paragraph).

## Conclusion

The findings of the present study show that plyometric exercise training performed on the tramp-trainer is highly effective for inducing rapid gains in muscle volume, architecture, torque, and power in healthy younger adults.

Notably, changes in VL volume and fascicle length were detectable already after 2 weeks of training, followed by increases in knee-extensors volume and power from week 4 of PLT.

## Data Availability Statement

The raw data supporting the conclusions of this article will be made available by the authors, without undue reservation, to any qualified researcher.

## Ethics Statement

The studies involving human participants were reviewed and approved by The University of Nottingham Ethics Committee and was compliant with the Declaration of Helsinki. The patients/participants provided their written informed consent to participate in this study.

## Author Contributions

MF, MN, and SL designed the study. EM, FB, JQ, and MF performed the experiments. EM and MF analyzed the data. EM, MF, SL, FB, JQ, and MN discussed the data. EM, MF, SL, and MN wrote the manuscript. All authors contributed to the article and approved the submitted version.

## Conflict of Interest

The authors declare that the research was conducted in the absence of any commercial or financial relationships that could be construed as a potential conflict of interest.

## Footnotes

1 http://imagej.nih.gov/ij/
